# Quantification of bone metabolic activity in the natural course of fractural lesions measured by quantitative SPECT/CT

**DOI:** 10.22038/AOJNMB.2022.63484.1446

**Published:** 2023

**Authors:** Tomohiko Yamane, Yohji Matsusaka, Kenji Fukushima, Akira Seto, Ichiro Matsunari, Ichiei Kuji

**Affiliations:** 1 Department of Molecular Imaging Research, Kobe City Medical Center General Hospital, Japan; 2 Department of Nuclear Medicine, Saitama Medical University International Medical Center, Japan; 3 Department of Radiology and Nuclear Medicine, Fukushima Medical University, Japan; 4 Division of Nuclear Medicine, Department of Radiology, Saitama Medical University Hospital, Japan

**Keywords:** Bone fractures, Single photon emission computed tomography, Radionuclide imaging, Quantification, Standardized uptake value

## Abstract

**Objective(s):**

While increased uptake at the bone fractural site gradually decreases over time on bone scans, the rate of change has not been quantitatively evaluated. The purpose of this study was to quantify the extent of bone metabolic changes in fractural lesions on bone SPECT/CT.

**Methods:**

We reviewed bone scans acquired by dedicated SPECT/CT and chose those scans in which quantitative SPECT/CT of the same range was acquired twice or more. We set the region of interest on lesions of bone fracture and degeneration, and measured the maximum standardized uptake value (SUV_max_). From the SUV_max_ of lesions and the interval between scans, a value for 30-day change in SUV_max_ was calculated as ∆SUV_max_30d. The relationship between preSUV_max_, SUV_max_ for the first scan of the comparison, and ∆SUV_max_30d was evaluated utilizing a linear least-squares method. Furthermore, we assessed the ability to differentiate between fracture and degeneration using receiver operating characteristics (ROC) analysis and the Mann-Whitney *U* test. All cases were then categorized into five groups according to preSUV_max_. Values of *p* <0.05 were considered statistically significant.

**Results:**

We investigated 175 scans from 60 patients and analyzed scan combinations for 157 fractural lesions and 266 degenerative lesions. The relationship between preSUV_max_ of fractural lesions and ∆SUV_max_30d was approximated as ∆SUV_max_30d =-0.15×preSUV_max_ +1.35 (*R*^2^=0.60, *p*<0.0001). Area under the curves for all cases, 30≤ preSUV_max_, 20≤ preSUV_max_ <30, 15≤ preSUV_max_ <20, 10≤ preSUV_max_ <15, and preSUV_max_ <10 were 0.73, 0.89, 0.86, 0.80, 0.91, and 0.59, respectively. Median ∆SUV_max_30d was significantly lower at fractural lesions than at degenerative lesions (-0.62 vs -0.04; *p* <0.0001). As for analyses of groups divided by preSUV_max_, all median ∆SUV_max_30d for fractural lesions were significantly lower than those of degenerative lesions except for the group with preSUV_max_ <10.

**Conclusion:**

The increased uptake at the fractural bone lesion observed in the quantitative bone SPECT/CT gradually decreased at the rate of SUV 0.15 per month, which showed a different trend with degenerative change.

## Introduction

 Bone scans show increased tracer uptake in fractural lesions. When evaluating bone metastasis, uptake in the fracture occasionally interrupts precise diagnosis, especially for solitary lesions (1). Accumulation at fractural sites is well known to gradually decrease (2), 

and this decline can be one of the keys to differentiating fractural lesions from malignant lesions. However, how fast the accumulation decreases over time has not been characterized objectively.

 Recent advances in single photon emission computed tomography (SPECT) have been improving quantitative technology (3, 4). In addition to software-based methods (5), hardware-based techniques combined with computed tomography (CT) can accurately calculate quantitative values (6). Standardized uptake value (SUV), a representative quantitative value used initially in positron emission tomography (PET), has also been used in SPECT quantification. Using SUV calculated from quantitative SPECT/CT systems, we can discriminate bone metastases from benign lesions (7). In addition, SUV from bone SPECT can be used to diagnose various benign diseases (8, 9) and evaluate physiological bone metabolism (10) as well as monitor the effects of treatment in cancer therapy (11).

 Clarification of quantitative changes in the bone metabolism of fractural lesions is essential to understand the pathogenesis of bone metabolism. The changes observed in fractures are expected to differ from those involved in degenerative lesions, which also often show positive accumulation on bone scans. The purpose of this study was therefore to describe the extent of bone metabolic changes in fractural lesions observed by bone scintigraphy.

## Methods

***Patients***

 The protocol for this retrospective study was approved by the institutional review board of Saitama Medical University International Medical Center (approval no. #20-056), and the need for written informed consent was waived. We reviewed all clinical images from bone scans acquired between August 2017 and April 2020 from a dedicated SPECT/CT platform (Symbia Intevo; Siemens, Erlangen, Germany). We included only those cases in which quantitative SPECT/CT of the same range was acquired at least twice. Among those, we excluded scans showing extravascular tracer leakage or insufficient image quality for the calculation of quantitative values. We also eliminated from analysis those scans showing no apparent fracture or degeneration. In addition, while we used two kinds of tracers for bone scans, scans that used different tracers were also avoided in the analysis.

***SPECT/CT and quantification***

 Patients received approximately 740 MBq of 

^99m^Tc-methylene diphosphonate (^99m^Tc-MDP) or ^99m^Tc-hydroxymethylene diphosphonate (^99m^Tc-HMDP). Approximately three hours after injection of the tracer, SPECT/CT images were acquired using the integrated SPECT/CT scanner (Symbia Intevo; Siemens, Erlangen, Gernamy). Parameters for SPECT were: continuous rotation mode; acquisition, 180° for each head with a non-circular orbit; energy peak, 140 keV with 15% width; projection, 60 views over 180° with a dwell time of 10 s/view (total, 10 min/bed position); reconstruction, ordered subset conjugate gradient minimizer algorithm with one subset and 48 iterations; preset mode with scatter and attenuation corrections, “enhanced” and “skeletal” (xSPECT Bone; Siemens); matrix, 2.54×2.54×2.54 mm; and pixel size, 256×256. Furthermore, parameters for the CT were: voltage, 130 kV; tube current, automatic dose modulation (Care Dose 4D; Siemens) with a set reference current of 60 mAs; tube rotation time, 0.6 s; pitch, 1.0; and matrix, 512×512.

***Image analysis***

 Two board-certified nuclear medicine physicians evaluated lesions showing increased uptake on SPECT images and chose those lesions considered to represent either fracture or degeneration by consensus. The physicians drew regions of interest (ROIs) on a workstation for nuclear medicine (syngo.via NM Oncology WF for VA 30; Siemens), and SUV was calculated by the following equation:

SUV=Activity concentration Bq/ml×Body weight (g)Injected radioactivity (Bq)

 In the analysis, SUV_max_ was used offering maximal SUV from the pixels within the ROI. The ROI was manually drawn on follow-up images in the same area as in the first scan, and SUV_max_ was measured.

***Statistical analysis***

 For a bone scan image in which a fractural or degenerative lesion was present, we examined all other follow-up scans in which the same lesion was observable and recorded the SUV_max_ of each lesion and the interval in days between scans. If scans were performed three times or more, data were recorded between all combinations, i.e., when three scans were performed, data were recorded three times; between the first and second scans, between the first and third scans, and between the second and third scans. In cases where abnormal uptake disappeared during follow-up, we did not include the case in subsequent analyses. To facilitate an understanding of the rate of change, standardized values for the 30-day rate of change

∆SUV_max_30d) were calculated using the following formula,

$\mathrm{SUVmax30d}=\frac{\mathrm{postSUVmax}–\mathrm{preSUVmax}}{\mathrm{days between the scans}}\times 30.$

where postSUV_max_ and preSUV_max_ indicate SUV_max_ at the later and earlier scans, respectively.

 We evaluated how much SUV_max_ of the day changed after 30 days utilizing a linear least-squares method on fractural and degenerative lesions by comparing the relationship between preSUV_max_ and ∆SUV_max_30d. In addition, we evaluated the ability to differentiate between fracture and degeneration by receiver operating characteristics (ROC) analysis and the Mann-Whitney *U* test. Areas under the curve (AUCs) were calculated for ROC analysis. These differential abilities were analyzed in all cases and the following five groups for preSUV_max_ were defined: 30≤ preSUV_max_, 20≤ preSUV_max_ <30, 15≤ preSUV_max_ <20, 10≤ preSUV_max_ <15, and preSUV_max_ <10. Values of *p *<0.05 were considered statistically significant. We used integrated statistical software (Prism 8.4.3; GraphPad Software, San Diego, CA, USA).

## Results

***Patients and analyzed lesions***

**Figure1:**
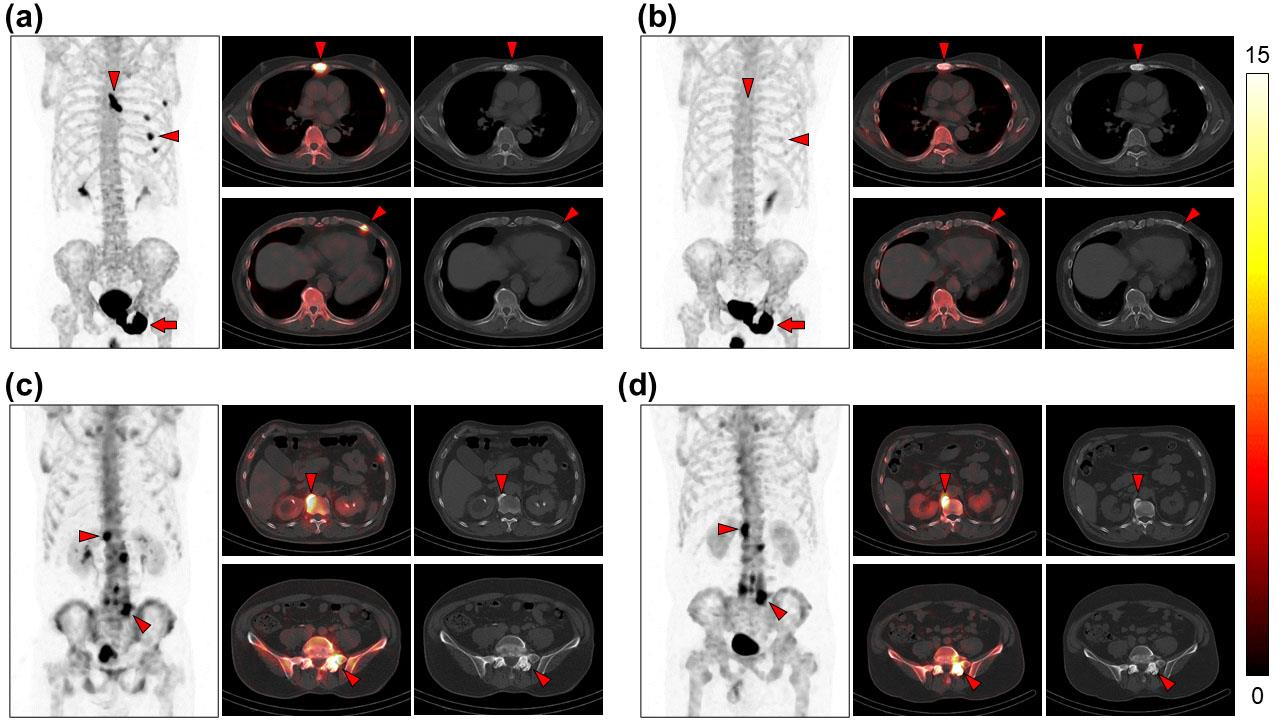
Maximum intensity projections, fused SPECT/CT, and CT images of representative cases; first scan (**a**) and the follow-up scan after 155 days (**b**) in fractural lesions, and first scan (**c**) and the follow-up scan after 169 days (**d**) in degenerative lesions. SUV_max _of bone fracture in the sternum changed from 30.0 to 6.2, and in the left fifth rib changed from 17.2 to 4.6 (**arrowheads**). Bone metastases are also visualized as intensively increased uptake in the pubis and ischium (**arrows**). SUV_max_ of degeneration in the Th12 osteophyte changed from 19.4 to 20.7, and in the left lumbosacral facet joint changed from 18.8 to 22.9 (**arrowheads**). The color scale in the fused SPECT/CT images is illustrated on the right side of this figure ranged SUV 0—15. SUV, Standardized uptake value; SUV_max_, maximum SUV

 Among patients who underwent bone SPECT/CT scan twice or more, we reviewed 666 bone SPECT/CT scans from 206 patients. A total of 19 scans met the exclusion criteria, comprising 18 scans with extravascular leakage of tracer and 1 scan with insufficient image quality. We also excluded a further eight scans in which paired scans were unavailable. Moreover, we excluded 464 cases in which no fracture or degeneration lesions were evident. As a result, we finally included and analyzed 175 scans from 60 patients in this study. Table 1 summarizes detailed patients characteristics. Among these, we identified 157 combinations for fractural lesions and 266 combinations for degenerative lesions. Table 2 summarizes the detailed characteristics of these combinations. None of these combinations of scans used different tracers. Figure 1 shows representative cases.

**Table 1:** Patient characteristics

**Number**		60
**Age** **(patient-based)**	Range, median	44–86, 73.5
**Sex** **(patient-based)**	Men	54
	Women	6
**Tracer for bone scan** **(patient-based)**	^*99m^Tc-MDP	54
	^**99m^Tc-HMDP	6
**Primary disease for bone scan** **(patient-based)**	Prostate cancer	52
	Breast cancer	4
	Renal cell carcinoma	2
	Gastric cancer	1
	Thyroid cancer	1
**Total lesions analyzed**	Fracture	66
	Degeneration	77

^* 99m^Tc-MDP, ^99m^Tc-methylene diphosphonate; ** ^99m^Tc-HMDP, ^99m^Tc-hydroxymethylene diphosphonate

**Table 2:** Characteristics of analyzed combinations between two scans

**Analyzed scan combinations**	Fracture	157
	Degeneration	266
**Interval between scans (days)**	Fracture (range, median)	24–822, 176
	Degeneration (range, median)	28–886, 261.5
**Fractural lesions** **(combination-based)**	Rib	122
	Vertebra	23
	Clavicle	6
	Pelvis	4
	Sternum	2
**Degenerative lesions** **(combination-based)**	Vertebra including osteophyte and adjacent joint	210
	Around joint in the chest area	56

***Regression Analysis***

**Figure 2:**
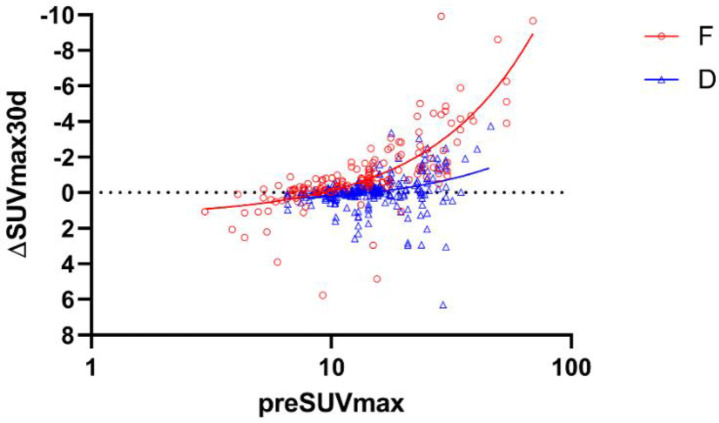
Scatterplot of lesion SUV_max_ on the first scan (preSUV_max_) and the rate of change in SUV_max_ standardized to 30 days (SUV_max_30d). Fractural lesions (**F**) are shown as red circles, and degenerative lesions (**D**) are shown as blue triangles. Red (**F**) and blue (**D**) lines illustrate linear regression. Horizontal dot line indicates SUV_max_30d=0. Note that the horizontal axis of preSUV_max_ uses a logarithmic scale

 Scatterplots of the relationship between preSUV_max_ and ∆SUV_max_30d are shown in Figure 2. For fractural legions, the formula was as follows:

SUVmax30d=-0.15×preSUVmax+1.35.

 With this formula, the 95%CI of the slope was -0.17 to -0.13, and the intercept ranged from 0.97 to 1.73. The *R*^2^ value was 0.60, and the *p*-value was <0.0001. This indicated that the fractural lesion that showed an initial SUV of 40 

displayed a decrease in SUV of 4.6 per 30 days, SUV=30 decreased by 3.1 per month, and SUV=20 decreased by 1.6 per month.

 For degenerative lesions, the formula was as follows: 

SUVmax30d=-0.04×preSUVmax+0.62.

 With this formula, the 95%CI of the slope was -0.06 to -0.03, and the intercept ranged from 0.33 to 0.92. The *R*^2^ value was 0.09, and the *p*-value was <0.0001.

***Differentiation between fracture and degeneration***

**Figure3:**
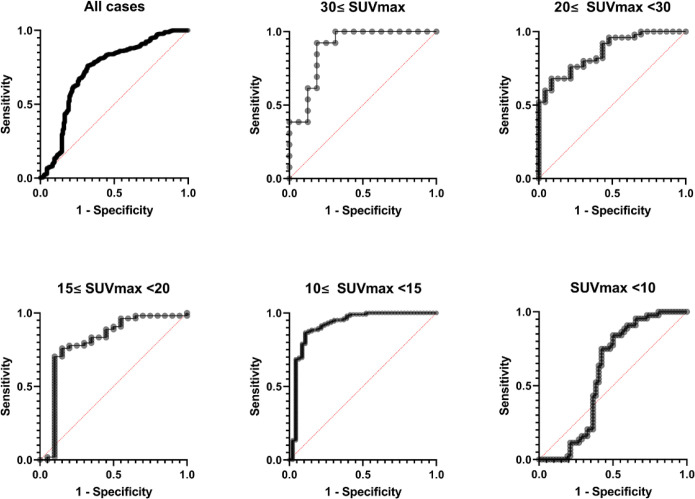
Receiver operating characteristic analyses to discriminate fracture from degeneration by ∆SUV_max_30d. Diagonal red dotted lines represent non-discriminatory tests

Figure 3 illustrates the ROC curves to differentiate fractural lesions from degeneration. Table 3 summarizes the AUC of the ROC and other statistical data for differentiating fractures from degeneration in all cases and in the five groups divided according to preSUV_max_.

**Table 3:** Summary of statistical data on differential ability by preSUV_max_

	**All cases**	**30≤ PreSUV**_max_	**20≤ PreSUV**_max_ ** <30**	**15≤ PreSUV**_max _ **<20**	**10≤ PreSUV**_max_ ** <15**	**PreSUV**_max _ **<10**
**Area under the curve (95%CI)**	0.73(0.67–0.78)	0.89(0.77–1.00)	0.86(0.78–0.95)	0.80(0.67–0.94)	0.91(0.86–0.98)	0.59(0.47–0.71)
**Median SUV**_max_ **30d ** **on fracture**	-0.62	-4.10	-2.24	-1.16	-0.60	-0.04
**Median SUV**_max_ **30d ** **on degeneration**	-0.04	-1.50	-0.33	-0.14	-0.03	0.10
***P*** ** values**	<0.0001	<0.001	<0.0001	<0.0001	<0.0001	0.14
**Number of fractures**	157	16	23	20	46	52
**Number ** **of degenerations**	266	13	50	54	105	44

## Discussion

 Utilizing quantitative SPECT/CT, we evaluated the natural course of bone metabolic activity in fractural and degenerative lesions using SUV_max_. We found that the SUV_max_ of fracture decreased significantly earlier than that of degeneration, which may offer a key characteristic for discriminating between these pathologies. The significance of the present study is that we quantitatively demonstrated the possibility of determining whether a lesion showing increased uptake represents fracture after a certain period when encountering such cases. 

 The uptake on fractural lesions gradually decreased at a constant rate, whereas degenerative lesions appeared to show relatively little change. Although not included in this study, malignant bone tumors, including 

metastases, without any treatment are expected to increase the uptake in a short period, further facilitating discrimination between them.

 We evaluated the rate in SUV_max_ over time and standardized the changes to 30 days as ∆SUV_max_30d. The ∆SUV_max_30d was significantly higher for fractural lesions than for degeneration, and these findings were consistently observed in groups with 10≤ SUV_max_. While no significant difference between values was identified for the group with SUV_max_ <10, such SUVs can be considered to reflect physiological or near-physiological uptake. 

 Inverting the approximate equation in fractural lesions, the ∆SUV_max_30d was estimated as around zero when preSUV_max_ was 9. A previous paper reported that the SUV_max_ of normal bone is approximately 5–10 (12-14), generally consistent with our findings.

 We can find a lot of positive numbers of ∆SUV_max_30d in Figure 2, which indicates uptake increase after follow-up, in fractures and degeneration in which SUV_max_ was around 10 or less. Such results may indicate that ∆SUV_max_30d is converging to zero. On the other hand, some combinations take an extremely high positive ∆SUV_max_30d among them. Such values may suggest that a fractural site has re-fractured and is showing even more increased accumulation. Once fractured, these fragile areas could be re-fractured by minor stressors such as coughing or trauma (15). 

 Detailed interviews with the patients are indispensable to understand the pathological condition. There seem to be more certain technical errors in the quantification of SPECT/CT than in PET, but these errors are generally considered within the permissible range (16). Moreover, Arvola et al. reported that SUVs of SPECT/CT and PET/CT with ^18^F-NaF are quite similar (12).

 We used 30 days for standardization because this is easy to understand as a quantitative value, but we should note that this study did not evaluate actual data from a 30-day period for reference. A more extended period may be required to assess changes, or determination over a shorter period may be feasible. Additional prospective studies are necessary to clarify the optimal timings in the future.

 We consider the present study had some limitations. First, final diagnoses were not based on pathological results, but rather on visual diagnoses by expert nuclear medicine physicians. Decisions in the present study could thus have included some misdiagnosed pathologies. Second, this study did not include any malignant bone lesions. Differentiating bone metastasis from benign fracture is clinically essential, but adding metastatic lesions into this kind of analysis would not be easy, because such metastatic lesions should be treated immediately. Modification of the uptake by treatment cannot be excluded in this analysis. Third, some of the combinations included relatively long periods between scans. 

 After a certain point, uptake stops changing, which would introduce errors into the calculation. In this study, the maximum interval between scans was 886 days, and we cannot exclude the possibility of underestimating the change in such cases. And finally, we evaluated several types of fractures together. The healing process of a fracture includes an inflammatory phase, a reparative phase, and a remodeling phase (17), and the tracer accumulation of bone scintigraphy varies according to these phases. 

 Therefore, it is important to evaluate separately by the exact injury time and the type of fracture or location.

## Conclusion

 Quantitative SPECT/CT depicted the natural time course of bone metabolic activity on fracture and degeneration by SUV_max_. 

 SUV_max_ decreased significantly earlier for fracture than for degeneration.
